# Universal Dependence of Nuclear Spin Relaxation on the Concentration of Paramagnetic Centers in Nano- and Microdiamonds

**DOI:** 10.3390/ma15165774

**Published:** 2022-08-21

**Authors:** Alexander M. Panich

**Affiliations:** Department of Physics, Ben-Gurion University of the Negev, Beer-Sheva 8410501, Israel; pan@bgu.ac.il

**Keywords:** spin–lattice relaxation time, spin–spin relaxation time, nanodiamonds, paramagnetic defects, concentration dependence

## Abstract

An analysis of our data on ^1^H and ^13^C spin–lattice and spin–spin relaxation times and rates in aqueous suspensions of purified nanodiamonds produced by detonation technique (DNDs), DNDs with grafted paramagnetic ions, and micro- and nanodiamonds produced by milling bulk high-temperature high-pressure diamonds is presented. It has been established that in all the studied materials, the relaxation rates depend linearly on the concentration of diamond particles in suspensions, the concentration of grafted paramagnetic ions, and surface paramagnetic defects produced by milling, while the relaxation times exhibit a hyperbolic dependence on the concentration of paramagnetic centers. This is a universal law that is valid for suspensions, gels, and solids. The results obtained will expand the understanding of the properties of nano- and microdiamonds and will be useful for their application in quantum computing, spintronics, nanophotonics, and biomedicine.

## 1. Introduction

Detonation nanodiamond (DND) particles are of significant scientific interest and are very promising materials for modern science and applications in quantum computing, spintronics, nanophotonics, and biomedical applications due to the small size of primary particles (5 nm) with a narrow size distribution, easy surface functionalization, high biocompatibility, and possibility of production in large quantities [1,2,3,4,5,6,7,8,9,10,11]. DND suspensions with grafted paramagnetic metal cations [12,13,14,15,16,17,18,19,20] exhibit a high relaxivity and are proposed as new contrast agents for magnetic resonance imaging (MRI) [16,17,18,20]. Recently, Sękowska et al. reported the possible use of DNDs in MRI phantoms [21] produced using distilled water, agar, and carrageenan with the addition of the DND particles suspended in dimethyl sulfoxide (DMSO). Surprisingly, the authors obtained linear dependences of the spin–lattice (*T*_1_) and spin–spin (*T*_2_) relaxation times in the phantoms as a function of the nanodiamond concentration (see Figures 3 and 5 in Ref. [21]). This result contradicts our recent experimental nuclear magnetic resonance (NMR) data on DND suspensions [16,17,18], as well as some fundamentals of relaxation phenomena in nuclear spin systems [22,23,24].

In this paper, we analyze the results of measuring proton and carbon nuclear spin–lattice and spin–spin relaxation times and rates in (i) aqueous suspensions of highly purified DNDs, (ii) aqueous suspensions of DNDs with grafted paramagnetic ions, (iii) powdered DNDs grafted with paramagnetic ions, and (iv) powdered micro- and nanodiamonds produced by milling bulk diamonds prepared by the high-temperature high-pressure (HTHP) method. We established that in all the studied materials the relaxation rates depend linearly on the concentration of nanodiamonds in suspensions, the concentration of grafted paramagnetic ions in suspensions and in powder samples, and surface paramagnetic defects produced by milling, while the relaxation times exhibit a hyperbolic dependence on the concentration of paramagnetic centers. This is a universal law that is valid for suspensions, gels, and solids. The results obtained will expand the understanding of the behavior of nano- and microdiamonds and will be useful for their applications in quantum computing, spintronics, nanophotonics, and particularly in biomedical applications.

## 2. Materials and Methods

We report the data on aqueous suspensions of purified DNDs and DNDs with grafted Gd^3+^ ions, powder DNDs with grafted Cu^2+^ and Gd^3+^ ions, and milled HTHP nanodiamonds of the SYP series. Sample purification, EPR, and SQUID impurities monitoring and preparation of aqueous suspensions of highly purified and de-agglomerated DND particles are described elsewhere [12,13,14,15,16,17,18,25]. The average DND particle size is ca. 4.5–5 nm as determined by dynamic light scattering (DLS), transmission electron microscopy (TEM), and atomic force microscopy (AFM) measurements [12,13,14,15,16,17,18,26].

Grafting of the nanodiamond surface with copper and gadolinium ions was made by mixing aqueous suspensions of nanodiamond particles with aqueous solutions of copper acetate Cu(CH_3_CO_2_)_2_ or gadolinium nitrate hexahydrate Gd(NO_3_)_3_⋅6H_2_O [12,13,14,15,16,17,18]. Dissociated metal cations (Cu ^2+^ or Gd ^3+^) in this mixture undergo ion exchange with hydrogen atoms of surface carboxyl groups and subsequent chemical bonding to the nanoparticle surface [12,13,14,15]. Thereafter, we call these materials Cu-DND and Gd-DND.

Submicron diamond powders of Syndia SYP series, manufactured by L.M. Van Moppes & Sons SA, Geneva, Switzerland, and hereafter identified by the denomination SYP, were produced by milling initial CDFS HPHT microdiamond crystallites with an average particle size of 100 µm, which resulted in several fractions with average particle sizes of 18, 30, 86, 130, 208, and 386 nm [27]. An additional laboratory purification stage was carried out to exclude ferro- and paramagnetic impurities from the commercial SYP samples.

The EPR study of all samples of purified DNDs shows the concentration of paramagnetic defects in the range of (4 ÷ 7) ×10^19^ spin/g [26,28,29,30,31,32,33], while in SYP NDs the concentration varied from 6.7 × 10^18^ to 3.3 × 10^19^ spin/g depending on the particle size [27].

It is well established that the surface of DND particles is terminated by hydrogen atoms forming hydrocarbon, hydroxyl, and carboxyl groups [30,31]. They are the source of ^1^H nuclear spins. ^1^H and ^13^C NMR measurements of powder samples were carried out at room temperature (T = 295 K) using a Tecmag (Houston, TX, USA) pulse solid-state NMR spectrometer and an Oxford superconducting magnet in an external magnetic field *B*_0_ = 8.0 T, corresponding to the ^1^H and ^13^C resonance frequencies of 340.52 and 85.62 MHz, correspondingly. ^13^C spin–lattice relaxation times *T*_1_ were measured using a saturation comb pulse sequence ($\frac{\pi }{2}$ pulse train) [34]. Magnetization recovery in measuring *T*_1_ was fitted by a stretched exponential $M(t)=M_{\infty }\{1-\exp[-{\left(\frac{t}{T_{1}}\right)}^{\alpha }]\}$, which is characteristic of the spin–lattice relaxation through paramagnetic defects [12,13,14,15,19,20,26,27,28,29,30,31,35]. Here, $M_{\infty }$ is the equilibrium magnetization, and the parameter *α* varies in the range of 0.5 *< α <* 1. ^13^C spin–spin relaxation times *T*_2_ were measured using the Hahn echo method.

^1^H NMR measurements of nanodiamond suspensions were carried out at a temperature of 310.1 K (37 °C). The ^1^H spin–lattice relaxation times *T*_1_ were measured using an inversion recovery pulse sequence [34], while the spin–spin relaxation times *T*_2_ were measured using a Carr–Purcell–Meiboom–Gill (CPMG) pulse sequence [36].

## 3. Results and Discussion

### 3.1. Suspensions of Purified DNDs and DNDs with Grafted Paramagnetic Ions

As is known, DND particles exhibit intrinsic localized paramagnetic defects: (i) P1 nitrogen paramagnetic centers distributed over the entire diamond core and (ii) unpaired electron spins of dangling bonds positioned mainly in the near-surface layer [26,28,29,30,31]. The total defect density in DND particles measured by the EPR method is around 6 × 10^19^ spin/g [26,28,29,30,31]. In DND suspensions, the relaxation of the proton nuclear spins of the solvent is accelerated, owing to the interaction of protons with unpaired electron spins of the aforementioned paramagnetic defects [16,17,18]. The contributions of the DND-inherent paramagnetic defects to the experimentally measured proton spin–lattice and spin–spin relaxation rates $R_{1}^{\exp}$ and $R_{2}^{\exp}$ in suspensions are described [16,17,18] by the second term of the equations

(1) $$R_{1}^{\exp}=\frac{1}{T_{1}^{\exp}}=\frac{1}{T_{1}^{solv}}+\frac{1}{T_{1}^{DND}}=R_{1}^{solv}+r_{1}^{DND}\times C_{DND}$$

(2) $$R_{2}^{\exp}=\frac{1}{T_{2}^{\exp}}=\frac{1}{T_{2}^{solv}}+\frac{1}{T_{2}^{DND}}=R_{2}^{solv}+r_{2}^{DND}\times C_{DND}$$

where $T_{1}^{solv}$ and $T_{2}^{solv}$ are the relaxation times due to the solvent, $T_{2}^{DND}$ and $T_{1}^{DND}$ are the spin–lattice and spin–spin relaxation times caused by paramagnetic defects of the nanodiamond particles, *C*_DND_ is the DND concentration, and *r*_1_ and *r*_2_ are the relaxivities defined as the slopes of the concentration dependences of $\frac{1}{T_{1}^{\exp}}$ and $\frac{1}{T_{2}^{\exp}}$. Here, $T_{1}^{solv}$ and $T_{2}^{solv}$ are the characteristics of the particular liquid solvent used and, therefore, are constant for all measurements.

**Figure 1:**
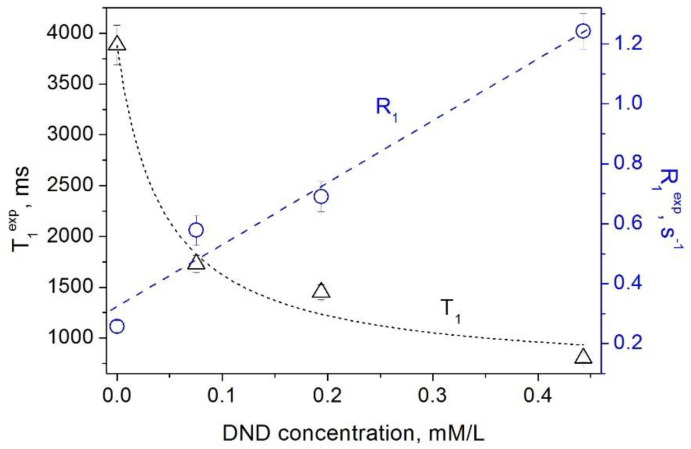
Dependence of the spin–lattice relaxation rate *R*_1_ (circles) [16] and the spin–lattice relaxation time *T*_1_ (triangles) of water protons in aqueous DND suspensions on the DND concentration.

**Figure 2:**
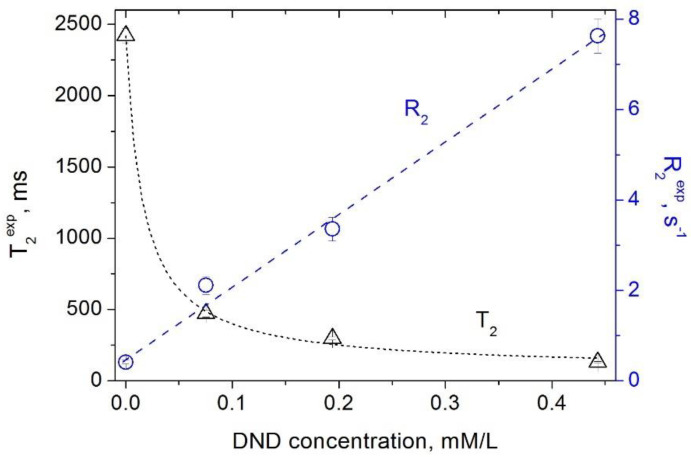
Dependence of the spin–spin relaxation rate *R*_2_ (circles) [16] and the spin–spin relaxation time *T*_2_ (triangles) of water protons in aqueous DND suspensions on the DND concentration.

The results of our measurements of the spin–lattice and spin–spin relaxation times and rates of water protons in aqueous DND suspensions as a function of the DND concentration are shown in Figure 1 and Figure 2. Our data show that paramagnetic defects of the DND particles (i) affect the relaxation rates of water protons in suspension and (ii) reveal a linear dependence of the relaxation rates $R_{1}^{DND}$ and $R_{2}^{DND}$ on the DND content. This finding is in accordance with the fundamentals of the spin relaxation theory [22,23,24], which demonstrates a linear dependence of the relaxation rate on the concentration of paramagnetic centers/defects. Herewith, as it follows from Equations (1) and (2) and the experimental data presented in Figure 1 and Figure 2, both proton spin–lattice and spin–spin relaxation times demonstrate a hyperbolic dependence on the concentration *C*_DND_ of nanodiamonds in suspension according to Equations (3) and (4):

(3) $$T_{1}=\frac{1}{R_{1}^{solv}+r_{1}^{DND}\times C_{DND}}$$

(4) $$T_{2}=\frac{1}{R_{2}^{solv}+r_{2}^{DND}\times C_{DND}}$$

This experimental result contrasts with the linear concentration dependence of *T*_1_ and *T*_2_ reported by Sękowska et al., [21]. The latter is inconsistent with that published in the literature and the fundamentals of the relaxation phenomena in nuclear spin systems, which casts some doubt on the correctness of the measurements and conclusions made in Ref. [21]. Herewith, we note that the measurements of Sekowska et al., particularly those of *T*_2_, were carried out in a limited range of nanodiamond concentrations, which causes some difficulties in establishing the character of the concentration dependence measured by these authors.

**Figure 3:**
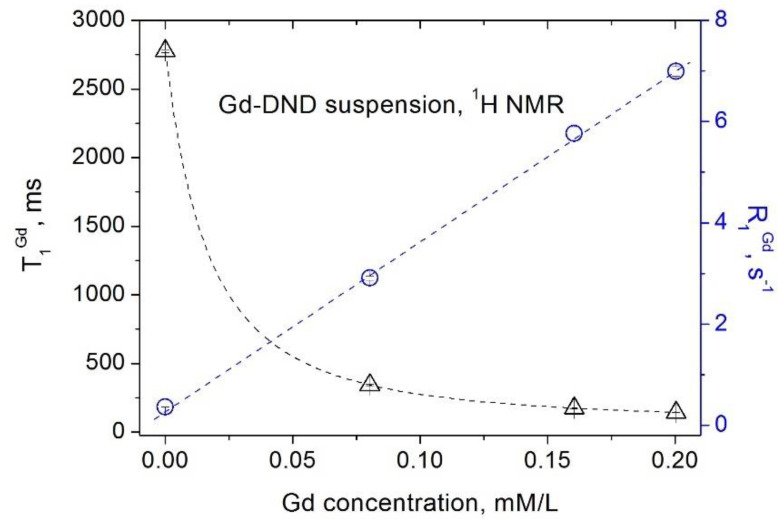
Dependence of the spin–lattice relaxation rate *R*_1_ (circles) [16] and the spin–lattice relaxation time *T*_1_ (triangles) of water protons in aqueous Gd-DND suspensions on the Gd^3+^ ion concentration.

**Figure 4:**
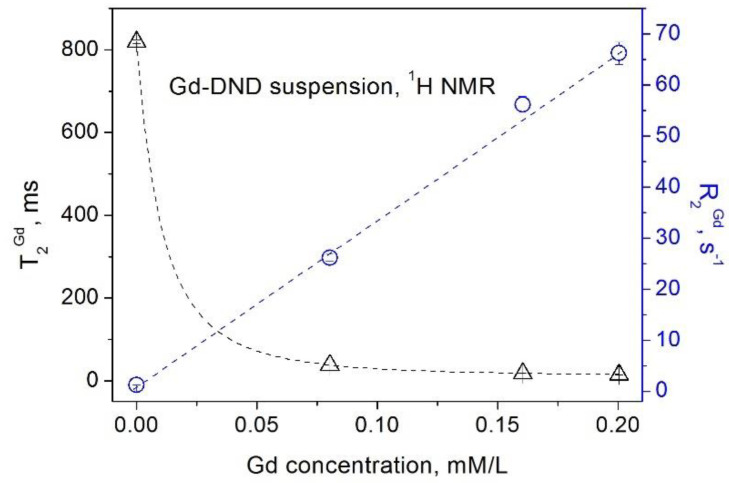
Dependence of the spin–spin relaxation rate *R*_2_ (circles) [16] and the spin–spin relaxation time *T*_2_ (triangles) of water protons in aqueous Gd-DND suspensions on the Gd^3+^ ion concentration.

Similar dependencies were obtained for suspensions of the gadolinium-grafted DND (Gd-DND), which are shown in Figure 3 and Figure 4. Contributions of paramagnetic gadolinium ions grafted to the DND surface to the spin–lattice and spin–spin relaxations of water protons in this case are:

(5) $$R_{1}^{\exp}=\frac{1}{T_{1}^{\exp}}=\frac{1}{T_{1}^{Gd}}+\frac{1}{T_{1}^{DND}}+\frac{1}{T_{1}^{H_{2}O}}=r_{1}^{Gd}\times C_{Gd}+R_{1}^{DND}+R_{1}^{H_{2}O}$$

(6) $$R_{2}^{\exp}=\frac{1}{T_{2}^{\exp}}=\frac{1}{T_{2}^{Gd}}+\frac{1}{T_{2}^{DND}}+\frac{1}{T_{2}^{H_{2}O}}=r_{2}^{Gd}\times C_{Gd}+R_{2}^{DND}+R_{2}^{H_{2}O}$$

where $C_{Gd}$ is the Gd(III) ions concentration in suspension.

Therefore, the proton spin–lattice and spin–spin relaxation times reveal a hyperbolic dependence on $C_{Gd}$:

(7) $$T_{1}^{\exp}=\frac{1}{r_{1}^{Gd}\times C_{Gd}+R_{1}^{DND}+R_{1}^{H_{2}O}}$$

(8) $$T_{2}^{\exp}=\frac{1}{r_{2}^{Gd}\times C_{Gd}+R_{2}^{DND}+R_{2}^{H_{2}O}}$$

We note that Gd(III) ions have a large unpaired electron spin of *S* = 7/2 and a large magnetic moment of 7.9 $\mu_{B}$ (here, $\mu_{B}$ is the Bohr magneton), thus their contribution to relaxation exceeds the DND contribution by more than an order of magnitude [16,17,18].

In addition to our data, we mention the measurements of an aqueous solution of the nanodiamond-polyglycerol-gadolinium(III) conjugate DND-PG-Gd(III) [37]. The relaxation rates *R*_1_ of water protons in this material show a linear dependence on the Gd concentration in magnetic fields of 1.5 T, 3.0 T, and 7.0 T.

### 3.2. Powder DNDs with Grafted Paramagnetic Ions

Similar dependences of the nuclear spin relaxation in nanodiamonds on the concentration of the paramagnetic ions were obtained in our measurements of powder samples. In this case, the spin–lattice relaxation rate $R_{1}=\frac{1}{T_{1}}$ of the nuclear spin *I*, which interacts with the unpaired electron spin *S* of the paramagnetic ion, is given by the expression [14,15,19,20,22,29,30,31]

(9) $$R_{1}(r_{})=\frac{1}{T_{1}(r)}=\frac{2}{5}\gamma_{S}^{2}\gamma_{I}^{2}\hbar^{2}S(S+1)[\frac{3\tau_{e}}{1+\omega_{I}^{2}\tau_{e}^{2}}](\frac{1}{r_{}^{6}})\times N_{S}$$

Here, *γ_I_* and *γ_S_* are the nuclear and electron gyromagnetic factors, *ω_I_* is the nuclear Larmor angular frequency, $r_{}$ is the distance from the nucleus to the paramagnetic ion, *τ_e_* is the correlation time of the electron spin of the paramagnetic ion, and *N*_S_ is the number of paramagnetic ions in the particle.

**Figure 5:**
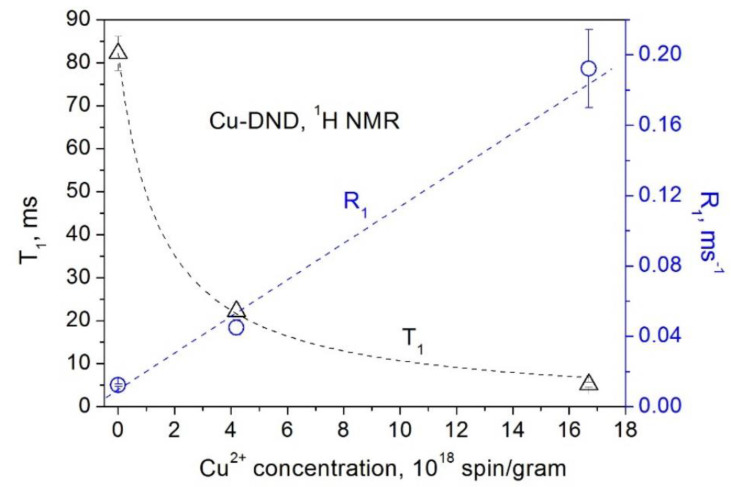
Dependence of the ^1^H spin–lattice relaxation rate *R*_1_ (circles) and the spin–lattice relaxation time *T*_1_ (triangles) in Cu-DND powders on the Cu^2+^ ion concentration.

**Figure 6:**
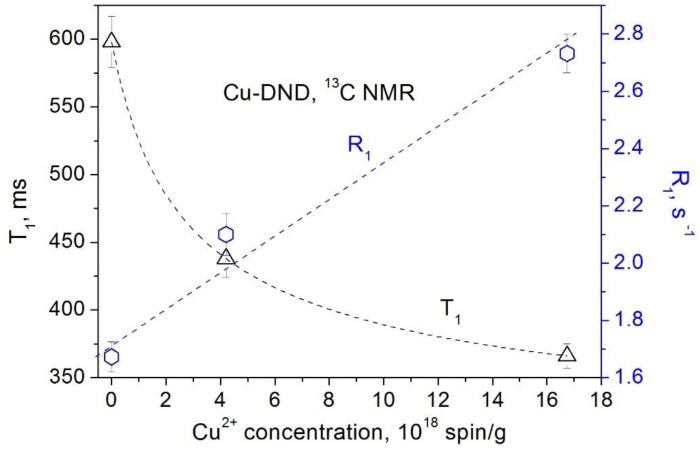
Dependence of the ^13^C spin–lattice relaxation rate *R*_1_ (circles) and the spin–lattice relaxation time *T*_1_ (triangles) in Cu-DND powders on the Cu^2+^ ion concentration.

**Figure 7:**
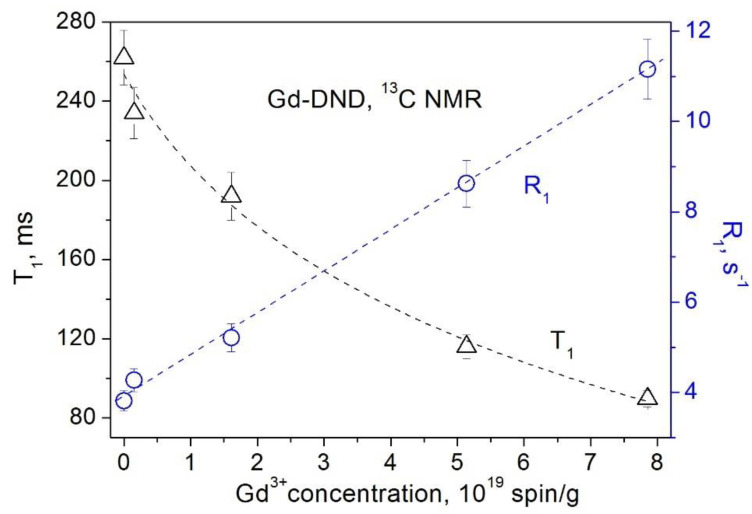
Dependence of the ^13^C spin–lattice relaxation rate *R*_1_ (circles) and the spin–lattice relaxation time *T*_1_ (triangles) in Gd-DND powders on the Gd^3+^ ion concentration.

**Figure 8:**
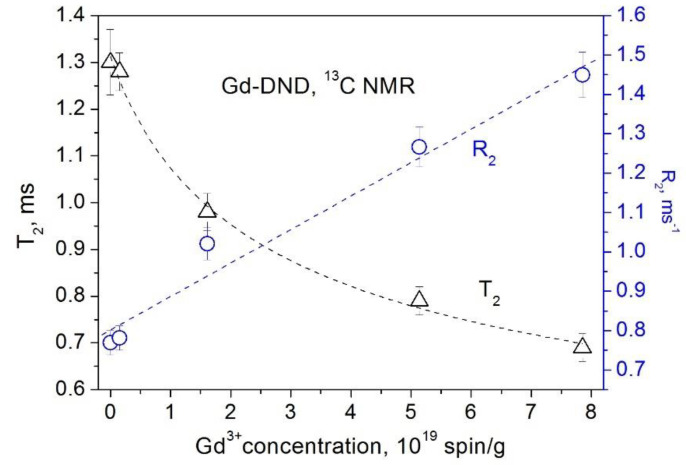
Dependence of the ^13^C spin–spin relaxation rate *R*_2_ (circles) and the spin–spin relaxation time *T*_2_ (triangles) in Gd-DND powders on the Gd^3+^ ion concentration.

The obtained dependences of the ^1^H and ^13^C spin–lattice relaxation times and rates on the concentration of paramagnetic Cu^2+^ and Gd^3+^ ions grafted to the DND surface are presented in Figure 5, Figure 6, Figure 7 and Figure 8. All these data show a linear dependence of the spin–lattice and spin–spin relaxation rates $R_{1}^{}$ and $R_{2}^{}$ and a hyperbolic dependence of the relaxation times $T_{1}^{}$ and $T_{2}^{}$ on the paramagnetic ions concentration. This finding is consistent with the fundamentals of the spin relaxation theory [22,23,24], which demonstrates a linear dependence of the relaxation rate on the concentration of paramagnetic centers.

### 3.3. Powder HPHT Nanodiamonds

**Figure 9:**
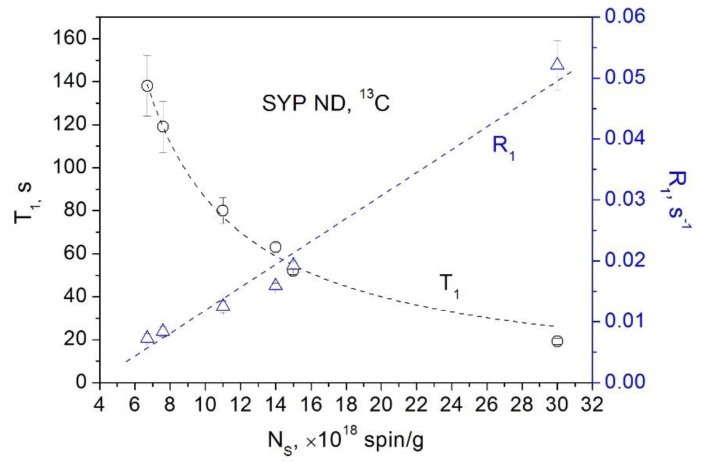
Dependence of the ^13^C spin–lattice relaxation rate *R*_1_ (triangles) and the spin–lattice relaxation time *T*_1_ (circles) in SYP nanodiamond powders on the concentration of the paramagnetic defects.

Let us move on to the powder nanodiamonds of the Syndia SYP series manufactured by L.M. Van Moppes & Sons SA (Switzerland) by milling the initial high-pressure high-temperature (HPHT) microdiamond crystallites with an average particle size of ∼100 μm. According to the size distribution datasheets provided by the manufacturer, this milling process provides several SYP fractions with average particle sizes of 18, 30, 86, 132, 208, and 386 nm, respectively, which were used in our study along with the initial SYP micro CDFS of the size of 100 μm. In Figure 9, we present our NMR measurements of SYP nanodiamonds of various sizes, in which the main contribution to relaxation is made by paramagnetic defects (mainly unpaired electron spins of broken bonds) associated with surface and subsurface defects that appear during the process of diamond milling [27]. Such paramagnetic centers produced by mechanical damage (e.g., milling) are often found in insulators and semiconductors, including diamonds, and are observed in EPR experiments [38,39,40,41]. On diminishing the average size of the ND fraction, the density of these defects increases from 7.6 × 10^18^ spin/g in the fraction of the largest particle size to 3.3 × 10^19^ spin/g in the fraction of the smallest particle size. Figure 9 clearly shows the linear dependence of the spin–lattice relaxation rate and the hyperbolic dependence of the spin–lattice relaxation time on the concentration of the paramagnetic defects in this series of materials.

Hyperbolic-like concentration dependence of *T*_1_ was recently obtained in measurements of the ^1^H spin–lattice relaxation of aqueous solutions of nanodiamonds of 18 and 125 nm in diameter prepared by the HPHT technique [42].

The data obtained in our measurements demonstrate the universality of the dependence of the nuclear spin relaxation in nanodiamonds on the concentration of the paramagnetic centers both in suspensions and in powder samples. This is a universal law that is valid for solutions, suspensions, gels, and solids [12,13,14,15,16,17,18,22,23,27,29,30,31].

### 3.4. Other Materials Containing Gadolinium Ions

The universality of the dependences of nuclear spin relaxation in nanodiamonds on the concentration of paramagnetic centers in both suspensions and powder samples obtained in our measurements is supported by data on other non-diamond lanthanide complexes promising for NMR/MRI diagnostic probes [43]. For example, proton relaxation rates for Gd_2_O_3_ nanodisks of different diameters and Gd-doped iron oxide nanoparticles of various sizes and shapes were measured in water after the nanoparticle surface functionalization with polyethylene glycol (PEG) dibasic acid. Both relaxation rates *R*_1_ and *R*_2_ reveal a linear dependence as a function of the Gd and Gd-Fe concentrations [44,45].

The relaxation rates *R*_1_ and *R*_2_ of water protons taken at room temperature in aqueous solutions of SiO_2_-coated quantum dots with grafted Gd-DOTA complexes at various concentrations ranging from 0.125 to 4 μM reveal a linear dependence on the Gd concentration [46].

Longitudinal relaxation rates and transverse relaxation rates as a function of concentration for aqueous solutions of gadolinium diethylenetriamine-pentaacetic acid (Gd-DTPA) and gadolinium DTPA-bis methylamide (Gd-DTPA BMA) at 23 °C represent a linear regression of the data, from which the relaxation rates *R*_1_ and *R*_2_ were determined [47].

The *R*_1_ values of Gadomer (Gadolinium-1,4,7,10-tetraazacyclododecane-*N,N’,N’,N’”* -tetraacetic-monoamide-24-cascade-polymer), RESOVIST, or Ferucarbotran (a mixture of Fe_2_O_3_ and γ-Fe_3_O_4_ nanoparticles with a size of ~5 nm in a carboxydextran matrix), and GADOVIST (C_18_H_31_GdN_4_O_9_) in bovine plasma, measured in a magnetic field of 1.5 T at 37 °C, show a linear dependence on the Gd concentration [48].

These results support well the above findings about the universality of the dependence of nuclear spin relaxation on the concentration of the paramagnetic centers both in suspensions and powder materials.

## 4. Conclusions

It has been established that the dependences of the nuclear spin–lattice and spin–spin relaxation times and rates in nano- and microdiamonds on the concentration of intrinsic paramagnetic defects, surface-grafted ions, and milling-induced paramagnetic defects reveal a universal behavior for both suspensions and powder samples. The relaxation rates show linear concentration dependence, while the relaxation times exhibit hyperbolic dependence on the concentration of paramagnetic centers. This is a universal law that is valid for solutions, suspensions, gels, and solids. The data obtained will expand the understanding of the behavior of nanodiamonds and will be useful for their applications in quantum computing, spintronics, nanophotonics, and biomedicine. In our opinion, this is particularly important for the use of the nanodiamond suspensions as contrast agents and phantoms for MRI [16,17,18,20,49].
